# Current management of the axilla in patients with clinically node-negative breast cancer: a nationwide survey of United Kingdom breast surgeons

**DOI:** 10.1186/1477-7800-4-4

**Published:** 2007-02-14

**Authors:** Lucy Mansfield, Isi Sosa, Roberta Dionello, Ash Subramanian, Haresh Devalia, Kefah Mokbel

**Affiliations:** 1 St. George's University of London, Blackshaw road, London, SW17 0QT, UK

## Abstract

**Background:**

Precise knowledge of axillary lymph node status is essential in the treatment of operable carcinoma of the breast. For many years, axillary nodal clearance (ANC) has been an integral part of the conventional management of early-stage breast cancer. During the last few decades the trend of these surgical procedures has been one of decreasing invasiveness in order to try and achieve a much lower level of morbidity. To help reach this improved level of treatment the concept of the sentinel lymph node (SLN) was utilized. Recent studies have shown that SNB can provide an accurate assessment of the axillary nodal status in clinically node negative patients, negating the need to remove the majority of the axillary contents and thus reducing morbidity. A recent meta-analysis of all the literature to date appears to reveal that the dual technique (blue dye and technetium-labelled sulfur) is the gold-standard for successful identification of the SLN in the context of early-stage breast cancer. We aim to highlight the on-going wide range of differing methods employed, and compare this to the gold-standard recommended guidelines.

**Methods:**

A questionnaire was devised to provide a snapshot overview of the current management of the axilla in patients with clinically node-negative T1 invasive breast cancer amongst UK beast surgeons in August 2006.

**Results:**

Of the 271 UK surgeons, 74 (27.3%) performed ANC as the initial management of the axilla in patients with clinically node negative T1 invasive breast cancer, 56 (20.7%) used axillary node sampling (not directed by sentinel node mapping) and a total of 141 (52.0%) used the technique of SNB, of which 50 (18.5%) used blue dye alone and 91 (33.6%) used a combination of blue dye and radioisotope.

**Conclusion:**

Despite the obvious advantages, our survey has revealed that the procedure is only used by 52% of British breast surgeons in this subgroup of patients (clinically node negative, tumour equal of smaller than 2 cm) most of whom have no disease within the axilla. The reasons for this include limited hospital resources and lack of surgeons training and accreditation and ARSAC license (nuclear medicine license).

## Background

Precise knowledge of axillary lymph node status is essential in the treatment of operable carcinoma of the breast for a number of reasons. In the first instance it permits the accurate staging of the cancer and aids in the selection of optimal adjuvant therapy. In fact it can be considered to be one of the most important prognostic indicators of disease outcome in women with early breast cancer [1,2]. Patients with histologically involved lymph nodes can anticipate a survival in the region of 50% [2]. For many years, axillary nodal clearance (ANC) has been an integral part of the conventional management of early-stage breast cancer. During the last few decades the trend of these surgical procedures has been one of decreasing invasiveness in order to try and achieve a much lower level of morbidity. To help reach this improved level of treatment the concept of the sentinel lymph node (SLN) was utilized. Since the introduction of sentinel node biopsy (SNB) in the mid 1990's, the optimal management of the axilla in breast cancer patients has become an issue [3]. Recent studies have shown that SNB can provide an accurate assessment of the axillary nodal status in clinically node negative patients, negating the need to remove the majority of the axillary contents [4,5]. In addition, the accuracy of SNB has been well documented, with recent large studies reporting false negative rates in the range of 2–5% [1,6-10]. The advantage of SNB in comparison to ANC is the significant reduction in long-term morbidity associated with the reduced level of tissue trauma [11]. Prospective observational studies have shown no increased incidence of axillary recurrence and confirmed the negligible morbidity associated with the SNB technique [9,12]. An up to date literature review reveals many studies are recommending SNB as the optimal management of the axilla in early stage breast cancer [11,13,19-21]. The aim of this nationwide study is to identify the current management of the axilla in patients with T1, clinically node negative breast cancers by UK breast surgeons. We aim to highlight the on-going wide range of differing methods employed, and compare this to the gold-standard recommended guidelines.

## Methods

A questionnaire was devised to provide a snapshot overview of the current management of the axilla in patients with clinically node-negative T1 invasive breast cancer amongst UK beast surgeons in August 2006. This was distributed to all breast surgeons listed in the Royal College of Surgeons database (n = 403). Three questions were proposed: initial procedure performed (ANC, axillary node sampling or SNB), the use of intraoperative frozen section or cytology and the further management plan if the SNB or axillary node sample was identified to contain tumour cells (radiotherapy versus ANC). Importantly we subdivided the initial question to identify how many surgeons used blue dye alone and how many utilized the dual technique (radioactive isotope and blue dye) in view of the current literature recommendations.

## Results

A total of 271 (67.2%) completed questionnaires were received. Of the 271 UK surgeons, 74 (27.3%) performed ANC as the initial management of the axilla in patients with clinically node negative T1 invasive breast cancer, 56 (20.7%) used axillary node sampling (not directed by sentinel node mapping) and a total of 141 (52.0%) used the technique of SNB, of which 50 (18.5%) used blue dye alone and 91 (33.6%) used a combination of blue dye and radioisotope. Amongst the surgeons who performed SNB using blue dye alone 26 also sent an additional four-node sample. 13 of the surgeons using the dual technique for sentinel node biopsy also did this.

With regards to the use of intra-operative examination, 7 surgeons (2.58%) used intraoperative frozen section to identify malignant cells within axillary lymph nodes, and 8 (2.95%) used cytological techniques. One surgeon used both frozen section and intraoperative cytology.

Amongst the 197 (72.7%) surgeons who did not undertake ANC as the initial management of clinically node-negative T1 invasive breast cancer, 124 (62.9%) proceeded directly to axillary node clearance if the sentinel node biopsy or four-node sample was positive for malignancy. However 31 (15.7%) completed treatment with radiotherapy alone. 8 (4.06%) used both axillary node clearance and radiotherapy. 33 (16.8%) used either axillary node clearance or radiotherapy dependent on a number of factors including extent of disease/number of positive nodes on axillary sampling, histological grade of the breast cancer, patient preference and general health/co-morbidities of the patient involved.

## Discussion

By definition, the sentinel node is the first node to which lymphatic drainage from the breast reaches. Thus these nodes are the most likely to harbor tumour cells if a breast cancer has indeed entered the lymphatics. These are usually located within the axilla, cranial to the intercostobrachial nerve, within 2 cm of the lateral edge to the pectoralis minor muscle [14]. However they may be found as an internal mammary node, a supraclavicular node or even a contralateral axillary node. The latter are very unusual. A tracer substance (a radioactive isotope, blue dye, and often both) injected into the breast provides a roadmap leading to the SLN(s). This follows the premise that the material (dye) will migrate through the lymphatics of the breast to the first lymph node(s) draining the tumour. These nodes are then removed and examined microscopically for the presence of metastatic tumour cells, often by frozen section techniques.

The SLN concept was initially introduced by Cabanas in 1977 [15]. He applied it to the management of penile cancer. The technique was popularised in the 1980s and early 1990s in the management of melanoma by Morton et al [16]. They used the injection of isosulfan blue dye to allow visualisation as it flowed through and stained lymphatic channels and nodes, enabling identification, excision and assessment of blue-stained nodes. In the early 1990s the SLN concept was applied to the management of breast cancer patients. These initial efforts followed the progress made in the management of melanoma, predominantly with the use of both radiotracer and blue-dye techniques to help identify the SLN. The first description of the use of SNB within the context of breast cancer was by Krag et al in 1993 [14].

International acceptance of the SNB over routine ANC is based on several considerations. In the first instance it is a much less invasive procedure that may be performed under local anaesthesia, often on an outpatient basis. It is well documented that it is associated with a much lower risk of common morbidities which are recognized features of full ANC. The results of the ALMANAC (a large prospective randomized control trial) support the use of SNB in patients with clinically node negative beast cancer in view of the benefits regarding arm functioning and quality of life [21]. In addition, the SNB allows the pathologist to study the few SLNs in greater detail compared with the larger number of lymph nodes removed in ANC. This leads to a greater degree of accuracy.

The SNB remains to be standardized universally; the methods, materials and patient selection vary by institution and surgeon. Identification of the SLN using 99 m-technetium-labelled sulfur was shown to be successful by Krag et al [3]. Giulian et al [14] demonstrated the same findings using blue dye and Albertini et al [17] used the dual technique, i.e. a combination of the two. Initial identification rates were reported at 82%, 66% and 92% respectively [3,14,17]. A recent meta-analysis of all the literature to date appears to reveal that the dual technique is the gold-standard for successful identification of the SLN in the context of early-stage breast cancer [18].

A study by Smidt et al from 1998 to 2003 [22] revealed that although axillary recurrences after a negative SNB do occur, this is at a much lower rate than would be expected on the basis of histological figures and the false negative SLN findings. The axillary recurrence rate in the literature is quoted as 0.25% [22]. Following review of the results of this study, the natural history of axillary relapse after negative SNB resembles the locoregional recurrence of breast cancer [22].

In comparison to the success of SNB, ANC appears to have a limited role in the initial management of early stage breast cancer. The majority (in the region of 70%) of women with clinically node negative axilla will prove to be microscopically negative as well [13]. As mentioned previously the ALMANAC study performed in the United Kingdom has shown that compared with standard treatment, SNB is quicker, does not require drain usage, is associated with a shorter hospital stay but more importantly, it significantly reduces the morbidity for the patient. The SNB is gradually becoming a new standard of care in patients with early breast cancer. However certain criteria should be fulfilled for its safe application. Despite the obvious advantages, our survey has revealed that the procedure is only used by 52% of British breast surgeons in this subgroup of patients (clinically node negative, tumour equal of smaller than 2 cm) most of whom have no disease within the axilla. This percentage is well below that reported in a similar study in the USA (74% in 2001 and likely to be much higher now) [23]. Of these American surgeons, 89% used both sulfur colloid radioisotope and isosulfan blue dye. This can be compared to only 33.6% (64.5% of those that perform the SNB) of British surgeons utilizing this optimal approach [24]. Intra-operative assessment of the sentinel lymph node is performed by a minority of British surgeons. This is most likely to be the consequence of limited resources. The implication of this finding is that a significant number of patients are undergoing a second surgical procedure if the SNB is found to contain malignant cells.

The reasons for the low percentage of British breast surgeons performing SNB include limited hospital resources and lack of surgeons' training and accreditation and ARSAC license (nuclear medicine license). The National Health Service (NHS) focuses on targets and the financial difficulties encountering most NHS hospitals are contributing to the low prevalence of the optimal application of this procedure. Hospitals and surgeons should therefore be encouraged to make the optimal technique of SNB available to their patients as a good alternative to ANC. The SNB in breast cancer training course (NEW START) is a unique team-based national training programme in the technique comprising theoretical teaching lasting one day and on-site proctored training. A validation series of 30 cases, which is audited centrally, is performed by each surgeon participating. Despite the figures produced from this survey, the prevalence of the SNB in the UK should improve over the next few years. NEW START has current data on over 2500 patients in the programme (all using isotope), thus there are 80–100 surgeons currently in training with an estimated 80% of British hospitals that have signed up for training but have yet all to complete their validation phase. The programme has now become international with training taking place in Norway and India, with visits planned for China next year.

Within this survey we did not investigate the use of routine preoperative lymphoscintigraphy to facilitate sentinel node identification. However recent studies [25] suggest that although it may be valuable for surgeons within the learning phase or in patients who have increased risk of intraoperative failed localisation (obese or elderly patients), this step is not essential to successful SNB given the time and cost required to perform this. In addition we did not examine the practice patterns of internal mammary dissection due to the lack of conclusive evidence that this procedure has a significant clinical benefit [26].

## Competing interests

The author(s) declare that they have no competing interests.

## Authors' contributions

LM was responsible for all literature reviews, data analysis and writing of the manuscript.

IS was responsible for design and distribution of the questionnaire.

RD was involved in data collection and analysis.

AS was involved in all literature reviews and editing of the manuscript.

HD contributed to the final editing of the manuscript.

KM conceived of the study and participated in its design and coordination, in addition to the final edit of the manuscript.
